# Wearable Sensing Devices: Towards the Development of a Personalized System for Construction Safety and Health Risk Mitigation

**DOI:** 10.3390/s21030682

**Published:** 2021-01-20

**Authors:** Chukwuma Nnaji, Ibukun Awolusi, JeeWoong Park, Alex Albert

**Affiliations:** 1 Department of Civil, Construction, and Environmental Engineering, The University of Alabama, 3043 HM Comer, Tuscaloosa, AL 35487, USA; 2 Department of Construction Science, The University of Texas at San Antonio, 501 W César E Chávez Blvd, San Antonio, TX 78207, USA; ibukun.awolusi@utsa.edu; 3 Department of Civil and Environmental Engineering and Construction, University of Nevada, Las Vegas, NV 89154, USA; jee.park@unlv.edu; 4 Department of Civil, Construction, and Environmental Engineering, North Carolina State University, 2501 Stinson Dr., Raleigh, NC 27607, USA; alex_albert@ncsu.edu

**Keywords:** wearable sensing device, safety technology, worker health, construction management, IoT

## Abstract

Wearable sensing devices (WSDs) are increasingly helping workers stay safe and healthy in several industries. However, workers, especially in the construction industry, have shown some aversion towards the use of WSDs due to their ability to capture specific information that may be considered personal and private. However, this revered information may provide some critical insight needed by management to plan and optimize worksite safety and support technology adoption in decision making. Therefore, there is a need to develop personalized WSD systems that are mutually beneficial to workers and management to ensure successful WSD integration. The present study aims to contribute to knowledge and practice by filling this critical gap using insight from 330 construction workers with experience using WSDs. The results from this study indicate that all 11 WSD functions identified through this study play a vital role in improving worker safety and health and that approximately two out of three workers are open to sharing the physiological and environmental information captured using these WSDs with their management. However, functions for detecting workers’ proximity to workplace hazards, specifically energized electrical materials, toxic gas, and fire/smoke, were the most critical functions that had mutual value to workers and management. Finally, the present study proposed and evaluated a phased personalized WSD system that should encourage successful WSD integration.

## 1. Introduction

Improving worker safety and health performance is a major issue that is of interest to stakeholders in the construction industry worldwide. This is because of the notoriety of the construction industry in terms of the number of fatal and nonfatal injuries recorded annually. The harsh and dangerous nature of the construction work environments leads to comparatively high rates of injuries, illnesses, and fatalities experienced due to falls from a height, musculoskeletal disorders, and being struck by objects [1]. Current safety practices, which are mainly passive in nature, have not yielded the desired optimum results. Further improvements are necessary to enhance construction safety through the implementation of innovative strategies, and existing studies indicate that wearable technology has the potential to improve workers’ safety and health. Existing methods used for detecting hazards or monitoring the safety behaviors of workers and the working environment have proven to be inadequate due to the level of subjectivity involved. In an effort to enhance safety performance in construction, researchers and industry practitioners have reported that the application of safety technologies within various phases of construction projects has the potential to significantly improve the safety and health of construction workers [2,3,4,5]. These technologies include exoskeletons, wearable sensing devices (WSDs), camera network systems, building information modeling, unmanned aerial vehicles, onsite task-specific robots, artificial intelligence, and immersive technologies (augmented and virtual reality). Specifically, these technologies could improve workers’ awareness of hazards, help eliminate hazards in the design phase, reduce work-related musculoskeletal disorders, facilitate accident investigation, enhance hazard visualization, and improve safety inspections [4,6,7,8]. WSDs have the potential to enhance worker safety through efficient data collection, analysis, and the provision of real-time information about safety and health risks to personnel [3,9,10]. The use of WSDs can open a new door toward occupational safety and health management in physically demanding and hazardous construction environments [11]. The current spread of wearable devices fitted with biosensor systems have provided an ample opportunity to continuously measure and understand workers’ physical demands from construction work [12].

The recent advancement in wearable technologies and big data technologies provides an accessible way to collect and transform safety and health data in real-time, which has great potential to improve safety performance by reducing injury-related risks [13]. Although studies posit that WSDs have huge potential to reduce injuries and fatalities and have recently started gaining attention [10,14], the existing literature suggests that the actual adoption, implementation, and extended use of the technologies in the construction industry have been slow [5,15]. The extended use of technologies in the construction industry is a complex phenomenon, impacted by multiple industry-specific variables. Previous reports in the construction industry have also highlighted the presence of some resistance to the use of WSDs [3,8]. To a large extent, this resistance impacts the outcome of safety technology integration [16].

The inherent concerns of workers with respect to the privacy and security of personal information have been recognized by researchers and practitioners as significant factors limiting the adoption and use of WSDs in construction [11,17]. However, this revered information may provide some critical insight needed by management to plan and optimize worksite safety and support technology adoption decision making. The potential benefits of wearable sensing devices for personalized safety and health monitoring in construction have been evaluated in several studies [3,8,18]. Therefore, there is a need to develop personalized WSD systems that are mutually beneficial to fieldworkers (FM) and management personnel (MP) to ensure successful WSD integration. The present study aims to contribute to knowledge and practice by filling this critical gap using insight from construction workers with experience using WSDs. The remainder of this paper is structured as follows: Section 2 provides background information, a WSD research overview, its functions, and applications in construction. Section 3 provides a comprehensive explanation of the materials and methods adopted in conducting this study. In Section 4, the results of the study are presented along with an extensive discussion of the findings, while Section 5 concludes the study and points to potential future work.

## 2. Background

### 2.1. WSD Research Overview

Wearable devices refer to any electronic device or product designed to provide a specific service that can be worn by the user [15]. They are electronic technologies or computers that are incorporated into items of clothing and accessories which can be worn comfortably on the body [19]. Unlike traditional wearable products, advancements in technology have made it possible to develop new wearable devices that are more sophisticated and can perform a wide variety of functions. Various types of wearable devices exist today, including smartwatches, fitness trackers, smart clothing, body sensors, and other wearable devices [15]. A few of these WSDs exist in the construction industry in the form of smartwatches, wristbands, smart hard hats, safety vests, smart boots, clips, tags, and so on [14]. Safety is one of the most critical challenges in the construction industry, which suffers from a high number of accidents (highest among all U.S. industries) [20]. These WSDs have been found to be very useful on construction sites for detecting near-miss falls or reducing fall-related injuries [10,21,22,23], identifying unsafe posture in workers and potential work-related ergonomic risks [9,24], monitoring workers’ fatigue and workload stress [18,25,26], and other applications. Many of these wearable devices are still in development and not commercially available. Some of the commercially available construction WSDs such as Spot-r Clips, EquipTags, SmartBoots, and Zephyr, combine various sensors into a single, compact, power-efficient platform.

The applications of WSDs and their impacts on construction safety and health have been extensively reviewed and investigated by several researchers. In one study, Choi et al. [11] investigated the determinants of workers’ adoption of wearable technology in the occupational work context. Workers’ intention to adopt two representative wearable devices (a smart vest with an embedded indoor GPS for location tracking, and a wristband-type wearable activity tracker with physiological sensors) for occupational safety and health were tested. The findings of this study provide insights into the role of various factors to motivate construction workers to adopt wearable technologies in their work. Awolusi et al. [3] and Anh et al. [8] conducted a critical review of the applications of WSDs in construction safety and health by identifying the general applications and functions, the applicable sensors and systems, the performance characteristics of WSDs, and the challenges impeding further development and deployment of WSDs applications. These studies provided recommendations on future opportunities for advancing WSDs applications in terms of multiparameter monitoring, sensor fusion, risk, and post-injury compensability assessments, as well as developing a business case for the application of WSDs in construction. Nnaji et al. [14] also investigated the impact of WSDs as a control measure for construction worker safety by showing how WSD functions could have prevented fatalities using archival data. They also assessed the perception of top management toward the use of WSDs. The findings of the study generated practical insights into how practitioners and technology manufacturers can improve the adoption and implementation of WSDs in the construction industry. The findings of these studies show that the appropriate utilization of wearable sensing devices can help mitigate and eradicate the incessant problem plaguing the construction industry.

Previous studies identified factors such as privacy risk, the security of information, and the safety of acquired data as concerns that impeded WSD adoption, implementation, and extended use in construction [3,11,17]. These concerns could be reduced by developing a personalized device that only transmits critical information. A recent study, contrary to traditional expectations, highlighted that most construction workers (in Finland) are open to sharing data targeted at identifying personal health risks, creating a safe work environment for colleagues, and improving overall workflow [27]. The study also noted that as a worker’s concern for data risk increases, their willingness to share data reduces. However, this study was conducted in Finland, which is known for being more open and progressive [28]. Moreover, the study did not assess workers’ openness to sharing data from specific functions of WSDs. Because workers are hesitant to give up their personal information, there is a need for a comprehensive investigation of worker-level perception [i.e., comparing the perceptions of field workers (FW) and management personnel (MP)] to obtain profound knowledge of perceptions toward WSD adoption in the construction industry.

### 2.2. WSD Functions and Applications in Construction

WSDs are unique in their requirements and functions because they incorporate computer and electronic technologies into clothing and other accessories [15]. These devices have the potential to positively impact construction workers’ safety and health. Studies indicate that WSDs can serve different functions and applications including physiological monitoring, environmental sensing, proximity detection, and tracking the location of a wide range of construction hazards and vital signals, which can provide early warning signs of safety issues to construction workers [3,8]. A few studies have evaluated the functions and applications of WSDs and how they can be used to effectively enhance worker safety and health. For instance, physiological data such as heart rate, breathing rate, body posture, etc., can be automatically tracked and analyzed using different sensors and systems to monitor workers’ fatigue, identify unsafe posture in workers, and prevent fall-related injuries [10,21,24,25,29]. In environmental sensing, hazardous gases, air quality, air particles, and possibly toxic chemical leaks, and inclement atmospheric conditions can be monitored on jobsites to provide early warning signals to construction workers [17,30]. WSDs for proximity detection are capable of providing real-time alerts to construction personnel and equipment operators during hazardous proximity situations [17,31,32]. These functions are instrumental in the design and implementation of safety policies, more personalized safety training, hazard identification, and enhanced safety supervision.

For technologies such as WSDs to be accepted by end-users in the construction industry, their value-adding impact must be identified, continuously evaluated, and established [17]. WSDs must provide information, in real-time, to the end-user and have the right functions in order for the implementation to be successful. The “right function(s)” is largely dependent on the end-users and beneficiaries of the WSDs (i.e., field workers and management staff). The need for personalized wearable monitoring has recently come to the forefront in construction and adjacent industries, which share some similarities [17,33,34], necessitating the need to explore the types of WSD function that workers are open to having and the minimum number and type of functions needed for the management to make informed decisions (and functions that justify investing in WSDs) before implementation [35]. It is, therefore, important to assess if the design of WSDs should incorporate functions that provide personal feedback of sensitive (or private) information, predominantly physiological metrics, to the primary users (i.e., workers) and non-sensitive information (such as environmental metrics) to secondary users (e.g., safety manager) so that appropriate actions and interventions can be taken by concerned parties to prevent potential injuries or accidents. This form of personalization of wearable monitoring may help alleviate workers’ resistance resulting from privacy concerns, thereby increasing the prospects of successful adoption, implementation, and extended use.

The existing research suggests that FWs are resistant to using WSDs primarily due to the functions that capture personal information such as heart rate [3]. This resistance could also impact how workers perceive the importance of physiological functions to a worker’s health and safety management. To develop a personalized system, it is critical to identify the functions that are most important to workers. As discussed above and illustrated in Table 1, WSDs have multiple functions. The three primary functions of WSDs in construction, along with a few examples as used in this study, are summarized as follows: (1) physiological monitoring—measurement and monitoring of workers’ body temperature, HRR/blood pressure, body posture, stress level; (2) environmental sensing—sensing smoke/fire, toxic gases or chemicals, noise level; (3) proximity detection and location tracking—detecting proximity to energized cables, falling object, heavy equipment or vehicle, open trench.

### 2.3. Research Objectives and Contributions

The present paper seeks to stimulate a broader academic discourse by: (1) identifying preferred types of WSDs in the construction industry; (2) identifying and assessing the impact of WSD functions on worker safety and health in the construction industry from the management and FW perspective; (3) investigating the disparity in openness to and the importance of data sharing between management and FWs; (4) proposing and assessing a framework for a personalized WSD system. The main contributions of this study are to (1) investigate the criticality of WSD functions within a construction safety and health context, and (2) propose a personalized WSD system for the continuous monitoring of workers’ safety and health and the provision of automated notification when at-risk conditions exist.

It is important to note that the analyses conducted in this study are not solely based on perception but are actual-use cases. The responses were drawn from individuals who were familiar with WSDs and have used these technologies on their jobsites, thereby reflecting insight from actual use and not just perception. This insight is more robust than complete reliance on the perception of use data.

## 3. Materials and Methods

### 3.1. Study Design

**Figure 1:**
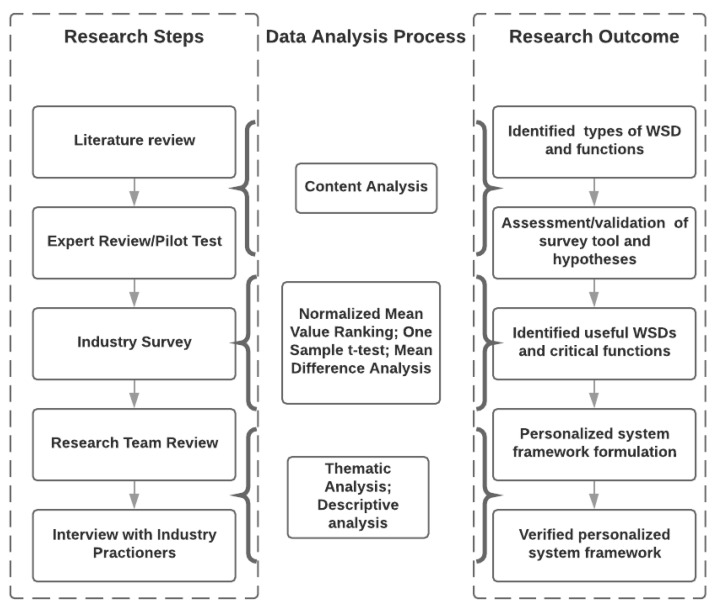
Research process and framework.

The methods used to investigate decision making regarding the use of technology could take multiple forms. However, the beliefs of the functions of a technology that are important could be perceived as a social construct, a subjective and emergent phenomenon, and should be investigated through discourse and interaction. Therefore, it is critical to adopt a pragmatist approach—a combination of Positivism and Interpretivism paradigm—in the present study, because quantitative and qualitative data, together, provide a better understanding of the fundamental research questions than either of the types independently. Hence, the present study implemented an exploratory mixed-method research approach that relies on the sequential collection of qualitative and quantitative data. It is expected that this method will ensure the aggregation of critical information needed to develop a framework for the personalized WSD system. It is important to note that the present study is part of a larger project focused on developing insight into the use of WSDs in construction safety management. The research process was divided into five research steps (Figure 1).

#### 3.1.1. Literature Review

The researchers conducted a review of the existing literature to formulate the research questions and hypotheses, and identify the different types of WSDs and their key functions, as well as factors that influence the integration of WSDs. Articles were extracted from academic databases such as Scopus and Web of Science. Given the emergent nature of academic research on WSDs [5], the researchers complemented academic articles with the grey literature on WSDs.

#### 3.1.2. Expert Review

As part of the research process, five individuals with industry and academic experience reviewed the research hypotheses and other findings from the literature review. The expert panel consisted of three professors and two project managers with construction-related experience ranging from five to 30 years. These individuals have intimate knowledge of construction safety practice and research. In addition, the experts participated in pilot testing the questionnaire survey to help ascertain the internal and face validity.

#### 3.1.3. Industry Survey

Based on findings from the literature review and expert insight, the researchers developed and distributed a questionnaire survey. The survey was designed to provide the information needed to identify WSDs used on construction projects, preferred types, important functions, and the types of data that workers are open to sharing. Participants were asked to select all WSDs that they have used on a construction project and rank the types of WSDs based on their preference of use (by indicating their most preferred (1) to their least preferred (11)). Using a 7-point Likert scale, participants were also asked to indicate the WSD functions that are most critical to workers’ safety on a jobsite and the WSD function data they want to see in real-time and those they are willing to share. The survey was distributed via Qualtrics. Given the emerging nature of WSDs and the limited use of the technology in the construction industry, and the need to ensure that adequate responses are received from a geographically diverse population, the researchers opted to distribute the survey using a third-party platform. Previous studies on WSDs have identified and sampled participants using the Qualtrics data management platform [36]. The researchers designed the study to achieve a minimum statistical power of at least 0.8. The power analysis was conducted for two-sample test, paired test, and one-sample analysis using a significance level of 0.05 and a medium effect size (0.5). The power analysis determined that a sample size of at least 100, 66, and 66 participants are required for two sample analysis, paired analysis, and one-sample analysis, respectively.

#### 3.1.4. Research Team Review

The fourth step in the research process involves the formulation and assessment of a framework for a personalized WSD system. In this step, the research team, which comprises five researchers with extensive experience in construction safety and technology use research (12 years of experience, on average), assessed the result from the survey and formulated a framework for a personalized WSD system that will have better odds of diffusion across the industry. This step will help ensure that multiple academic perspectives are provided for the framework. Insights from the academics will be synthesized and consensus will be gained before moving to the next step.

#### 3.1.5. Interview with Industry Practitioners

In the final step, the framework for a personalized WSD system will be presented to several construction workers to assess the usefulness, feasibility, and applicability of the personalized WSD system, and identify recommendations for improvements. In this phase, the researchers will interview multiple industry practitioners. Importantly, practitioners with varying experience will be afforded the opportunity to review the proposed personalized system and provide valuable feedback. Subsequently, participants will be asked to rate the final system. The assessment and feedback received from participants will equip the research team with the knowledge needed to modify and improve the recommended framework for personalized system. Additionally, opportunities and future research needs will be identified through discussions with industry personnel (field and management personnel).

### 3.2. Data Analysis

#### 3.2.1. Content Analysis and Thematic Analysis

Content analysis is a research tool used to detect and describe the presence and frequency of keywords, themes, or concept in qualitative data such as text [37] while thematic analysis provides a qualitative and detailed insight into the themes identified in a given dataset [38]. Data retrieved from querying databases were reviewed using content and thematic analysis, which are recommended tools for analyzing the output of a literature review and interviews [39,40].

#### 3.2.2. Descriptive Statistics

The present study relied on descriptive statistics to rank factors considered in the study. For instance, the count and frequency function was used to rank the types of WSDs used on construction projects and to identify the number of workers open to sharing data. Mean and standard deviation (SD), the most commonly used descriptive statistics [41], were used to rank the perceived impact of WSD function on workers’ safety. If two or more WSD functions have equal mean values, the function with the smaller SD was ranked higher [42]. Mean normalized value (MNV), which is also referred to as a relative analysis of criticalities, is used to determine the criticality of factors [43]. WSD Functions with MNV values/scores above 0.5 are deemed critical. Similar to the mean value, if two or more WSD functions have the same MNV, the function with the smaller SD is ranked higher.

#### 3.2.3. One Sample *t*-Test

To test the significance of the mean value of the impact of WSD functions on workers’ safety and health, the present study adopted the one-sample *t*-test. A *p*-value < 0.05 suggests that the factor is important, while a *p*-value above 0.05 suggests the factor is not important. Previous studies have adopted the one-sample *t*-test to test the significance and importance of green technology promotion strategies [44], barriers to implementing sustainable practices [45], leadership characteristics [46], and barriers to innovation [47].

#### 3.2.4. Mean Difference Analysis

The researchers adopted paired and two-sample *t*-tests to assess intra- and inter-group differences, respectively [48,49]. The paired *t*-test was used to compare the difference in the mean value of the level of importance of WSD functions between physiological monitoring and environmental sensing. This test was conducted for FWs and MP. The two-sample *t*-test was used to compare the mean value of the importance of WSD function between FWs and MP. The analysis also included the comparative analysis of the physiological and environmental functions of these groups. All assumptions for using parametric analyses were met.

#### 3.2.5. 2 × 2 Matrix Analysis

To help identify the most promising and valuable WSD functions, the researcher utilized an adapted version of the importance-performance (IPA) 2 × 2 matrix system. This matrix utilizes a four-quadrant system to compare the relationship between two critical factors [50]. This study uses the matrix approach to analyze the relationship between the impact of each WSD function and workers’ openness to sharing data with management. The description for each quadrant and a detailed explanation of the analysis process is provided in Section 4.4.4.

## 4. Result and Discussion

### 4.1. Characteristics of Participants

**Figure 2:**
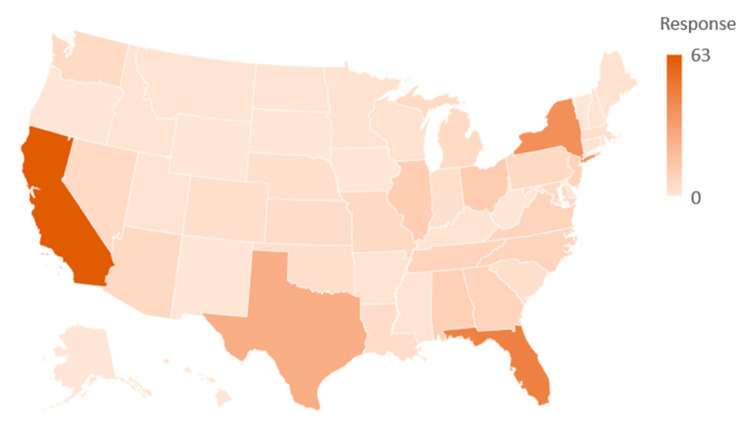
Response distribution across the US.

The survey was completed by 330 participants from across the US. California recorded the most responses (62), followed by Florida (46), New York (39), and Texas (26). The collated responses included at least one response from 80% of states in the US. Figure 2 depicts the spread of responses received. Eighty-nine percent of responses received were from individuals who worked for general contractors, while 83% of respondents have at least five years of experience working in the construction industry. As shown in Table 2, 238 respondents either had an associate degree or a bachelor’s degree. Fifty-seven percent of responses are from management personnel, while 13 percent of responses came from carpenters.

### 4.2. Types of WSDs Used on Construction Projects

Survey participants were asked to indicate which type(s) of WSDs they have used on construction projects. Table 3 summarizes the responses from participants. As shown in Table 3, smartphone-based wearables and smart hard hats are the most used WSDs in the construction industry (84% and 72%, respectively). As described earlier, Smartphone wearables refer to the use of applications in a smartphone as a tool for monitoring environmental conditions such as noise level and reporting unsafe exposure to workers. While the use of smartphones as a WSD limits the need for workers to carry and use additional devices, it also comes with some safety risks. For instance, the use of cellphones could cause distractions that lead to accidents. While the Occupational Safety and Health Administration (OSHA) lacks a specific regulation pertaining to the use of cellphones, OSHA could cite an employer if a worker’s use of a cellphone introduces new hazards or amplifies existing hazards (violating the General Duty Clause). To avoid such citations and encourage the healthy use of cellphones, organizations should develop and implement cellphone policies. It is recommended that organizations should consider other types of WSDs that have less potential to cause distractions.

### 4.3. Ranking of WSDs Based on Workers’ Preference

**Figure 3:**
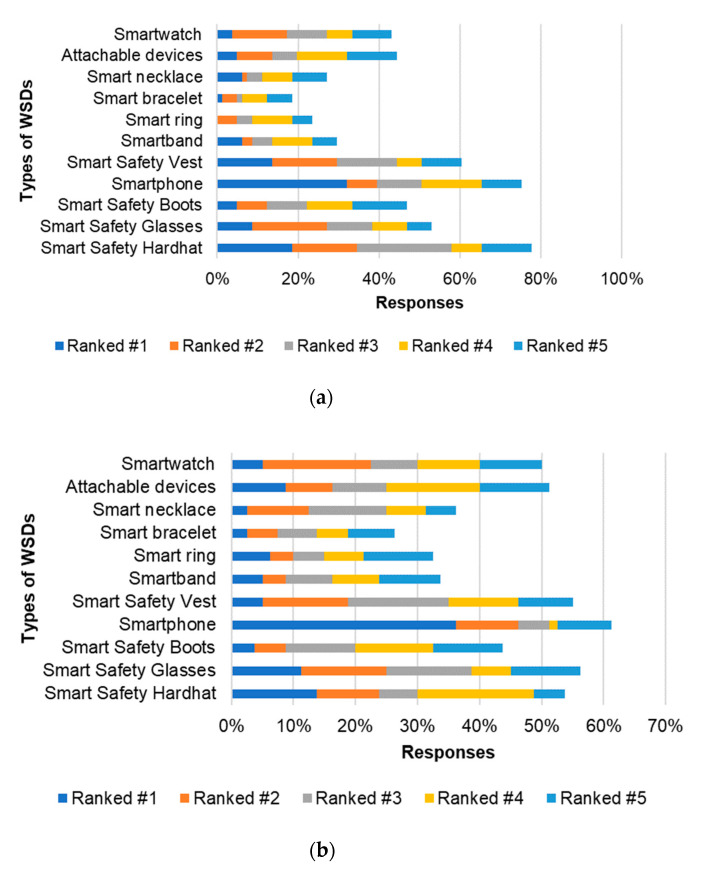
WSD Preference (**a**) FW (*n* = 140); (**b**) MP (*n* = 185).

As discussed in Section 2, and illustrated in Table 3, several types of WSDs are currently commercially available. However, workers are likely to have preferences because some of the designs and mounting positions of the WSDs on the body may raise concerns for workers. It is important to provide organizations with insight regarding the preferred types of WSDs to ensure that appropriate purchase decisions are made. Given the envisaged and projected increase in the manufacturing and supply of WSDs, identifying the preferred type of WSDs could inform the design of future WSDs, since manufacturers could use this insight to shape and inform the final design of their products. Therefore, participants were also asked to indicate their preferred types of WSDs. Figure 3a,b depicts the responses received from FWs and MP, respectively. Figure 3a,b, consistent with Table 2, ranks smartphone-based wearables as the widely used and most preferred WSDs (ranked as the most preferred by 32% and 35% of FWs and MP, respectively). This indicates that management and FWs are more comfortable with smartphone-based wearables. The preference for smartphones could be due to the reported resistance workers have against putting on additional PPEs or carrying additional devices [5,11]. The second and third most preferred WSDs (in terms of WSDs ranked #1) for FWs was the smart hard hat and smart vest. Smart hard hats and smart glasses received the second and third most first-place preferences by management.

When assessing WSDs that were ranked either #1, #2, or #3 by MP, smartphone-based WSDs remained the most preferred (51%), followed by safety glasses (39%) and safety vests (34%). However, for FWs, the smart hard hat was ranked higher than smartphone-based WSDs when aggregating their first, second, and third preference (58% and 50%, respectively). This was closely followed by safety vest (47%). Results from the descriptive analysis suggest that there is a difference between the preference of management and FWs. While FWs prefer WSDs in the form of smartphones, smart hard hats, and smart vest, MP prefer smartphone-based WSDs, smart glasses, and smart safety vest. As discussed in Section 4.2, the use of cellphones could significantly increase workers’ distraction. Therefore, management should endeavor to purchase smart hard hats and smart safety vest WSDs, given that FWs, who are the primary workers that utilize WSDs on construction projects, prefer these types of WSDs in the absence of smartphones. Additionally, manufacturers and researchers developing WSDs should shy away from cell phones. Researchers should focus on developing devices that are integrated into PPEs such as safety vests and hard hats.

### 4.4. Role of WSD Functions on Workers Safety and Openness to Data Sharing

#### 4.4.1. Importance of WSD Functions to Worker Safety and Health

Eleven WSD functions, available in the literature and commercially, that capture important data for improving worker health and safety, were identified in Section 2. However, there have been diverging opinions with regards to the usefulness of these functions in enhancing workers’ safety and health. Therefore, each participant was asked to assess the role these functions play in improving workers’ safety and health using a 7-point Likert scale. Table 4 contains the mean values, SD, MNV, and one-sample *t*-test. The mean values ranged between 5.39 and 5.92. According to Won et al. [51], mean values above 4 on a 7-point scale suggest the items measured are important, while item criticality could be determined by assessing the relative importance, MNV in this case [43]. This suggests that FWs and management believe that each WSD function can play an important role in improving their safety and health. “Smoke/Fire detection” and “Proximity to energized cable” are considered the top two useful WSD functions. It is important to note that while each physiological measuring function was rated above 5.0, none is ranked in the top five for FWs and MP (most physiological functions are ranked in the bottom three). This is likely due to workers’ resistance to the capturing of their personal information [11]. Interestingly, limited studies have empirically assessed the effectiveness of WSDs at detecting smoke/fire, energized cable, and toxic gas. In fact, most studies have focused primarily on assessing WSDs’ ability to assess and report health-related information such as stress, temperature, and fatigue/heat rate [8,17]. Therefore, researchers should increase investigations targeted at these environmental sensing functions. For instance, researchers could focus on investigating the effectiveness of energized system detectors such as Compass [52] and Proxxi [53] or developing advanced miniature technologies for accurately detecting and reporting voltage and current in real-time. Such studies should include recommendations on how to safely implement this group of devices; for example, providing a resource that highlights favorable construction operations, weather conditions, among others, will provide much-needed guidance and standards for WSD use in the industry.

To verify the level of importance of each WSD function, a one-sample *t*-test was conducted. The null hypothesis, H_0_, of this analysis is that “the mean value is not statistically significant,” whereas that of the alternative hypothesis, H_a_, is that “the mean value is statistically significant.” H_0_ should be rejected if a *p*-value is less than 0.05. The one-sample *t*-test was conducted by comparing the mean values to the mid-point of the scale (4, in this case). As shown in Table 4, all functions are significant (*p*-value < 0.05), thereby confirming that participants believe that each WSD function is significantly important to enhance their safety and health. The one-sample *t*-test result for FW and MP reflected an identical result (*p*-values < 0.05).

In terms of the criticality of these WSD functions, results from the normalization analysis suggest that FWs and MP believe that “Smoke/Fire detect,” “Proximity to energized cable,” “Toxic gases or chemicals detection,” and “Proximity to potential falling object are the most critical WSD functions (MNV above 0.5). In addition to these four WSD functions, MP believes that functions focused on warning workers when a vehicle is within close proximity are critical to workers’ safety and health (MNV = 0.78). Interestingly, no physiological function made the list of relative critical WSD functions from FWs’ and MP perspective. This result suggests that while physiological functions play a major role in keeping workers safe and healthy on a jobsite, construction workers believe that the environmental functions are more important to their safety. Section 4.4.2 provides a cluster-based analysis using inferential statistics.

#### 4.4.2. Cluster-Based Analysis

A close examination of the information provided in Table 4 suggests a disparity in how participants perceive the usefulness of physiological and environmental functions. Moreover, an assessment of data from FWs and MP suggests there is a potential difference in each groups’ perception of the usefulness of each WSD function. However, the significance of these differences should be assessed. As mentioned previously, it is important to identify and evaluate these differences to ensure that critical information covering multiple key perspectives vital to the successful implementation of WSD is provided. To assess if the differences are significant, a paired *t*-test was conducted to verify if the perceived importance of WSD functions were different across use (physiological monitoring or environmental sensing), while two-sample *t*-tests were conducted to assess the difference between FWs and MP.

In line with the existing literature, as described in Section 2, and the results postulated in Section 4.4.1, the present study posits that FWs believe that the environmental functions of WSDs have a significantly higher impact on worker safety and health relative to physiological functions. The hypotheses are as follows:

- H_0_ = mean value difference between environmental functions and physiological functions is equal to zero for FWs (µ_environmental − physiological_ = 0);
- H_a_ = mean value of environmental functions is significantly higher than the mean value of physiological functions for FWs (µ_environmental − physiological_ > 0).

First, the research team combined and averaged the mean values of all physiological functions to arrive at a single mean value (i.e., averaged the mean value for monitoring skin temperature, heart rate, body posture, and stress level, for instance). The same process was repeated to obtain a single mean value for environmental functions. While the average mean rating (importance) of environmental functions was higher (5.54) than physiological functions (5.45), there was no statistically significant difference between the perceived importance of physiological functions and environmental functions in improving the safety and health of a worker (*p*-value = 0.24, upper-tailed). Therefore, the null hypothesis was retained. This finding suggests that although environmental-based functions are rated higher than physiological functions in terms of their ability to improve worker safety, organizations looking to improve worker safety and health could adopt either category of functions or select a group of functions that meets their specific and/or immediate needs.

Next, the researchers evaluated the potential disparity in MP’s perception of which category of functions will be more useful to workers’ health and safety. The research team posits that management believes that physiological and environmental functions have equal impact on workers safety and health:

- H_0_ = mean value difference between environmental functions and physiological functions is equal to zero for MP (µ_environmental − physiological_ = 0);
- H_a_ = mean value difference between environmental functions and physiological functions is not equal to zero for MP (µ_environmental − physiological_ ≠ 0).

The average rating of importance for physiological functions and environmental functions was 5.80 and 5.86, respectively. Although the mean value of environmental functions was higher, the difference is not significant (*p*-value = 0.21). Therefore, the null hypothesis is retained, and it is concluded that management does not have any preference for physiological or environmental functions in terms of its perceived level of importance to enhancing workers’ safety and health. Management is satisfied if the WSD is collecting or providing information that improves workers’ safety and health. Although management believes that the functions are equally important, it is critical for managers to understand workers’ perspectives on the importance of physiological and environmental functions. Technologies, such as WSDs, rely heavily on FWs’ involvement and engagement (i.e., bottom-up implementation).

A primary factor that influences the adoption and successful integration of a technology is ensuring that the technology meets a critical need of end-users. The results from assessing FWs and MP responses indicate that all functions are impactful. Therefore, organizations integrating WSDs with the sole purpose of improving workers’ safety and health, with limited emphasis on data collection through WSD, should prioritize WSDs functions that meet their needs. For instance, a construction company in a region with extended periods of high temperature and humidity along with a high electrocution incident rate could opt to implement a WSD that captures body temperature and detects energized materials. Workers will be open to using WSDs with these functions. However, workers may be less likely to share data captured through physiological function (body temperature, in this case). It is important to note that this finding may differ if assessed with a pool of workers with no experience using WSDs. For instance, while workers with experience using WSDs find value in the physiological functions, workers with limited experience using WSDs will be less likely to find value in physiological functions due to their resistance to sharing their physiological data [27].

Following the paired *t*-test, a two-sample *t*-test was conducted to assess the potential difference in FWs and MP’s assessment of the importance of physiological and environmental functions to workers’ safety. Previous studies suggest that there might be a disconnect with how stakeholders assess the factors that influence the adoption of safety technologies in the construction industry [16,54]. However, a unified understanding of critical factors (WSD functions in this case), across key stakeholders, is critical to the successful implementation of a technology. The following hypotheses were formulated based on the existing literature:

- H_0_ = Mean values for physiological function impact are equal among FWs and MP;
- H_a_ = MP mean value for physiological function impact is significantly higher than FWs;
- H_0_ = Mean values for environmental function impact are equal among FWs and MP;
- H_a_ = MP mean value for environmental function impact is significantly higher than FWs.

Physiological functions: MP rated the average importance level of each physiological function 5.81 on a 7-point scale, while FWs rated the importance of physiological functions to their safety and health as 5.45. Results from a two-sample t-test showed a significant difference in the rating of importance between MP and FWs (*p*-value = 0.02). Therefore, the study concludes that MP believe that physiological-based functions could improve worker’s safety and health at significantly higher levels than are perceived by FWs.

Environmental functions: Similar to physiological functions, MP rated the perceived importance of environmental functions to enhancing workers’ health and safety higher than FWs did (5.87 and 5.53, respectively). Again, the difference between both groups was significant (*p*-value = 0.02). While each group rated the importance of environmental functions highly, management believes these functions can improve workers’ safety significantly more than workers’ current perception.

Furthermore, when the mean value of the perceived importance of physiological and environmental functions are combined, the average mean value for MP and FW are 5.82 and 5.49, respectively. Results from performing a two-sample *t*-test present an outcome similar to those reported above. That is, MP ranked the importance of WSD functions higher than FW (*p*-value = 0.01). The researchers conclude, from these results, that MP have a significantly stronger belief in the important and critical role WSDs functions play in keeping workers safe and healthy. Management, in certain cases, could be more optimistic than actual end-users with regard to the utility of a new technology. However, in certain cases, management’s optimism is supported by prior knowledge or existing data. If managements’ reason for being overly optimistic is supported by existing data, management should endeavor to provide FWs with insight on how the technology can improve workers’ safety and overall organization safety culture. Providing such information during safety training could raise awareness and generate a much-needed impetus to increase buy-in from FWs. Management could also identify and work closely with key fieldworkers such as foreman. Previous studies suggest that influential foremen and superintendents play vital roles in shaping the safety culture of a job site since they typically influence safety behavior at the most fundamental level—the crew level [55]. However, if management’s perception of the impact of a WSD on worker safety and health is indeed inflated, management should recalibrate expectations in line with reality. It is important to note that this disparity between MP and FWs could be more significant if FWs have no experience using WSDs.

#### 4.4.3. Data Sharing

Previous studies that investigated workers’ openness to sharing personal data (e.g., physiological data) showed that workers are typically unwilling to share their data [56]. Nevertheless, workers may be more open to sharing this data if the data are used for their own wellbeing [27,56]. The present study asked respondents to indicate if they are willing to receive the information generated from each WSD function and if they are comfortable with sharing data from each function with safety personnel. Respondents were asked to choose from the following options: receive information alone or receive the information and share with safety personnel. Additionally, participants were asked to ignore the question if they neither want to receive notifications provided by any function nor give access to safety personnel. MPs were asked to respond to the same question by indicating which functions they believe workers will be willing to share and those that FWs will prefer to keep private. Providing information from both perspectives would help identify if there is a perceived misalignment between FWs’ openness to data sharing and management’s preconception of workers’ openness to data sharing. By providing insight into workers’ data-sharing propensity, and the potential deviations between both groups, an organization could objectively re-strategize—ignoring preconceived biases—by placing emphasis on functions with greater value and acceptability to FWs and management. Table 5 summarizes the result from the FWs and MP perspective.

As shown in Table 5, the number of respondents who want to receive and share information from these functions with a safety professional on their project is higher than those who do not want to share data. Approximately two out of three construction workers with experience using WSDs are open to sharing WSD data with their employers (safety personnel, to be specific). This is a significant finding because previous studies concluded that most workers will not be open to sharing data [11]. However, the present study concludes that most workers—especially those with experience using WSDs—are willing to share data captured by physiological and environmental functions. Therefore, managers should encourage workers with experience using WSD to share their experience with workers with limited experience using WSDs. This could help reduce the resistance some workers have with regard to data sharing.

When comparing participants’ openness to sharing physiological and environmental data, surprisingly, FWs and MP indicated that they are more open to sharing physiological data than environmental data (FWs = 4.3%; MP = 1.1%). While workers rated environmental functions higher than physiological functions in terms of impact on worker safety and health, workers are more open to sharing physiological data. In certain cases, sharing data via environmental functions requires location-tracking devices. For instance, to determine the proximity of a worker to an excavated trench or a suspended object, it is important to know the worker’s location, since the trench and the location of the object are likely fixed while the worker is mobile. A primary concern that construction workers have with most WSDs is its ability to track worker’s location. Therefore, it is critical to explain to workers the benefit of collecting such information to ensure that they understand the value of location-based information. A closer examination of the data shows that this preference is not statistically significant (*p*-value = 0.06 and *p*-value = 0.12, respectively). This finding suggests that, contrary to popular expectations, the type of WSD function is not a key determinant in workers’ decision to share data.

Furthermore, results from comparing FWs and MPs’ willingness to share data suggest that MPs underestimated FW’s openness to sharing physiological data by 2.61%. For instance, 72.5% of workers are open to sharing information on their body temperature. However, MPs were under the impression that only 61.75% of workers will be willing to share body temperature data. This underestimation is likely due to the extensively documented expectation that workers will be less willing to share physiological data [27,55]. This result, while going against most expectations, provide more evidence that FWs are actually more open to sharing physiological data than they are given credit for—especially those with experience using WSDs. Therefore, construction managers and an organization’s top management should endeavor to limit assumption-driven technology integration decision making by assessing the state of their workers—what they are willing to share and what they prefer to keep private—prior to deciding what information must be collected.

#### 4.4.4. WSD Function Impact-Willingness to Share Analysis

While all 11 functions were perceived to be useful and important to worker safety (mean value between 5.39 and 5.92 on a 7-point scale), it is paramount to determine which functions, at the minimum, must be included in a WSD to encourage successful integration. To determine the functions with the most value to FWs and MP, the researchers utilized a 2 × 2 matrix analysis. The matrix was developed using MNV listed in Table 4 and described in Section 3.2.2, and the percentage of workers willing to share WSD data with management (Table 5). These values represent technology effectiveness and acceptability—critical factors that influence the adoption and implementation of technologies [16,53]. The threshold for determining acceptable MNV was 0.5, as described in Section 3.2.2. The researchers opted to utilize a 65% threshold for data sharing, which represents the approximate average data-sharing rate in this study (two of out three workers are open to data sharing). The Four Quadrants are explained below:

- Quadrant I (top right): This quadrant represents functions that are both impactful and acceptable. Functions highlighted in this quadrant should be prioritized by organizations, since they provide mutual value to organizations. This quadrant can be referred to as the “must-have/primary” quadrant;
- Quadrant II (top left): Given the critical role data sharing plays in WSD acceptance, and in line with the popular IP Matrix [50], the top left quadrant was identified as the second most important quadrant. Moreover, each function was rated above 5.0 with regards to the function’s impact on workers’ safety and health, which suggests that all functions are useful. Therefore, the matrix analysis is skewed towards prioritizing data sharing. Functions in this quadrant are important and should be given serious consideration. This quadrant is the “important/good to have/secondary” quadrant;
- Quadrant III (bottom right): This quadrant captures WSD functions that are important to workers’ safety and health but encounter slightly higher resistance to sharing data (workers sharing percentage between 50% and 65%). Functions in this category are important and should be reassessed after a period to determine if more workers are willing to share data generated from these functions. This is the “study and filter” quadrant;
- Quadrant IV (Bottom left): WSD functions in the last quadrant have a relatively lower impact on workers’ safety and health and more resistance to sharing data. While functions in this quadrant may be considered the least important functions based on the parameters used in developing the matrix in the present study, it is important to note that these functions could be relevant—depending on the context. This could be referred to as the “incorporate last” quadrant.

**Figure 4:**
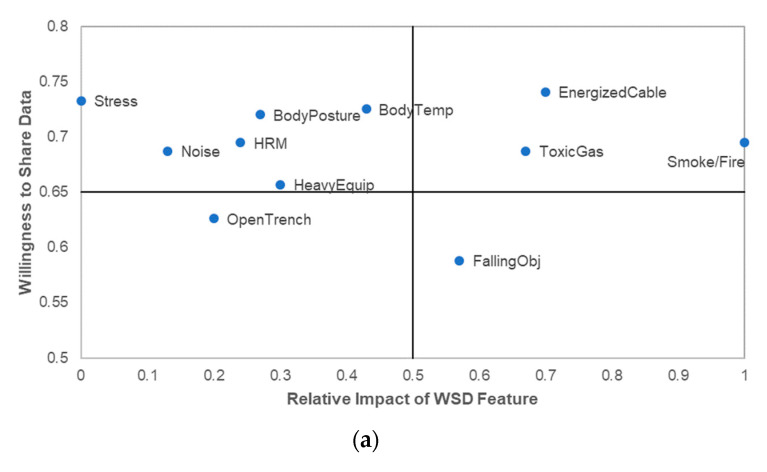

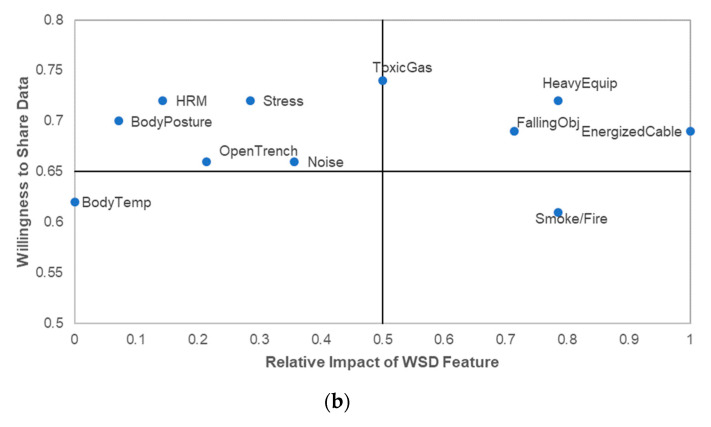
WSD function Impact–Willingness to Share 2 × 2 Matrix (**a**) FW; (**b**) MP. The researchers modified the position of some nodes to help with visual clarity. However, these changes did not distort the position of the functions relative to their respective quadrants, thereby ensuring that interpretation was not impacted.

The WSD functions that will be easiest to integrate into construction safety management are those that are present in Quadrant I for FWs and MPs. Figure 4a,b show the distribution of each WSD function into the four quadrants. These figures show that three WSD functions are present in FWs’ Quadrant I, while four functions are present in MP Quadrant I. A close inspection of these quadrants indicates that only two functions—“detection of energized cable/equipment” and “detection of toxic gas”—are present in Quadrant I of each group. FWs and MP believe that these functions are very impactful and that workers are willing to share data from these functions. Given that Quadrant I represents “must-have” functions, organizations that are integrating WSDs should prioritize WSDs with these functions. Moreover, this finding should inform the development of personalized WSDs. Manufacturers and researchers involved in the development of WSDs should deepen exploration associated with developing devices with these functions, thereby providing organizations with more effective and useful products.

Interestingly, all physiological functions (apart from “body temperature” for MP) are captured in Quadrant II. Their relatively high position in the 2 × 2 quadrant suggests that these functions could be incorporated within a personalized system depending on the context and the required threshold for safety and health impact.

Following the survey data cleaning and analysis, the research team reviewed the outcome of Section 4.4 to further evaluate and merge the findings. For instance, the research team opted to add “smoke and fire detection” to the list of critical functions, increasing the functions from two to three. The rationale behind this adjustment is that FWs indicated that they were open to sharing data on smoke and fire detection with safety personnel. Given that the FW group contains those most impacted by data sharing, any function that they are predominantly comfortable sharing should be considered. Therefore, the “smoke and fire detection” function is considered a critical function because it is ranked highly (impact on worker safety and health) by both groups and FWs are predominantly open to sharing data generated through this function. In addition, the research team incorporated some qualitative insights generated by participants that may be relevant to the development of a personalized WSD framework.

### 4.5. Proposed Personalized WSD System

The present study indicates that most FWs (>65%) are already conversant with three types of WSDs—smart hard hats, smart safety vests, and smartphones—and prefer using these devices relative to the other types of WSDs. While these devices could function independently as a wearable technology, smartphones also provide a platform for integrating other wearable technologies. Therefore, the smart safety vest and smart hard hat could be used independently or as a part of a system that includes a smartphone. Regardless, the selected smart device should be incorporated with sensors to capture information on proximity to energized equipment, toxic gases, and smoke/fire.

**Figure 5:**
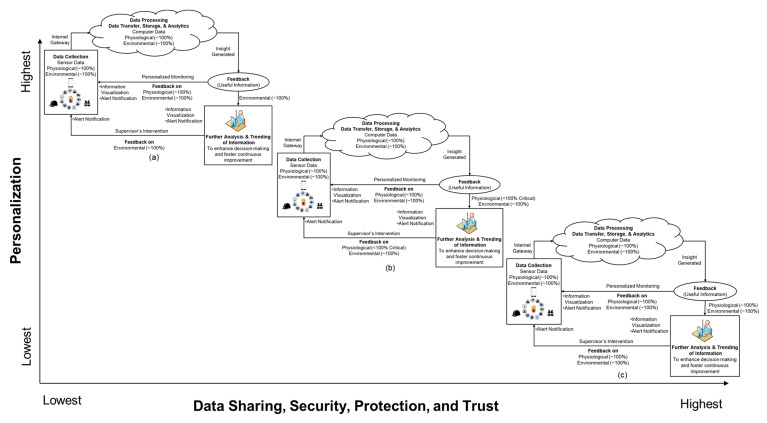
Framework for Personalized WSD System Transitionary Phases (**a**) limited data sharing; (**b**) increased data sharing; (**c**) 100% data sharing

Previous studies, and the present study, indicate that gradually phasing in technologies, such as WSDs, is the most pragmatic approach towards technology integration. The present study posits that while all WSD functions assessed in this study are important to worker safety and health, workers are predisposed towards using certain functions at this time. When combined with concerns associated with data security [17], data sharing [57], and trust [11], it becomes imperative that organizations consider personalized systems as a strategy to combat these challenges and increase acceptance. The present study proposes a framework consisting of three phases for progressively integrating WSDs into construction organizations. The framework depicted in Figure 5 shows the schematic of the proposed personalized system.

The framework typifies the transitionary phases of a personalized WSD system—starting with the three critical functions and expanding to all 11 functions. The first phase of the framework (Figure 4a) shows the proposed system, which is based on FWs receiving environmental and physiological notifications, while the safety professional receives only a select number of environmental metrics. For instance, if a worker is close to an energized cable, the wearable device transmits all information (both physiological and environmental metrics) to the fieldworker, while only the non-personal information (i.e., environmental metrics) will be transmitted to the safety supervisor. Figure 4b depicts a personalized system where the FW could control the type of information that is transmitted to the safety personnel. In the Figure, for instance, a worker could receive a warning from all 11 WSD functions. However, the safety personnel can only receive notification from the environmental functions (e.g., detection of energized cable/equipment, toxic gases, and smoke/fire) and specific physiological functions that the worker is willing to share. Importantly, there will be no penalty for refusing to share information from the other functions. Figure 5c depicts the third phase of integration, where workers and safety professionals could have access to data from all WSD functions. Workers receive alerts from all the functions and the same alert is transmitted, in real-time, to the safety professionals managing the specific project the worker is involved in. Sharing a wide range of information will provide safety personnel with the opportunity to provide personalized responses and feedback to workers.

The avalanche of data will provide an opportunity for an organization to develop robust predictive models for proactive safety and health management. It is expected that as time goes by and workers understand the value of the information being shared with their safety managers and other colleagues, workers will be more open to sharing data [56]. It is expected that the need for a personalized system will reduce significantly, as the IoT continues to diffuse across the industry, information technology infrastructure supporting the implementation of WSD becomes more robust and secure (introduction of standards and increased interoperability) as trust in WSDs increases.

### 4.6. Research Findings and Personalized WSD System Verification.

The final step of the research involved interviewing construction stakeholders. The objective of the interview was to provide potential end-users of the proposed personalized WSD system with an opportunity to provide contextual feedback on the research products. This validation step is considered critical because it provides additional internal and external validity [58]. According to Walsh [59], it is important to verify ethnographic research using respondent validation and triangulation. Moreover, this step acts as a verification and provides the researchers with an opportunity to integrate more qualitative perspectives, thereby increasing research robustness.

The research team developed an interview protocol that contained a summarized description of the research objectives and outputs/key findings. The protocol included information on the preferred type of WSDs, descriptive statistics on the importance of WSD functions, results from the *t*-tests, and the proposed WSD system. Interviewees were asked to rate their level of agreement for each key outcome using a 1–10 scale, where 1 represents “totally disagree,” 5 signifies “neutral” and 10 represents “totally agree” (Table 6). The researchers asked interviewees to evaluate the proposed personalized WSD system based on six criteria listed in Table 7. In addition, interviewees were given the opportunity to provide verbal or text-based responses. Furthermore, peer researchers within the safety engineering management domain were asked to review and verify the adequacy of the research process. Potential interviewees were purposively sampled to ensure high-quality response and response rate [60]. Participants were either sent a copy of the interview protocol via email, and interviewed in person, or through a phone call.

Ten construction stakeholders participated in the validation phase of the study. The researchers interviewed four project engineers, three project managers, one safety manager, and two construction safety researchers. The amount of construction experience ranged between one year and 20 years, while the median construction safety research experience was 10 years. Fifty percent of the participants were familiar with WSD.

Table 6 and Table 7 summarizes interviewees’ perceptions of the outcome of the study. As shown in Table 6, the median value of responses received from participants ranged between 6.5 and 9.5, which reflect participants’ relatively high level of agreement with the key research processes and outcomes. Participants believe that each key WSD function was covered in the present study, workers will be willing to share data from the four critical functions, and that management would likely have a higher regard for WSD functions. Moreover, the adequacy of the research design and statistical analysis was ascertained. This insight provides an additional level of verification.

In addition, participants were asked to rate the feasibility, usefulness, ease of implementation, and comprehensiveness of the proposed personalized WSD system. They were also asked to indicate the potential level of resistance from workers and management to personalize WSD system. Table 7 indicates that the participants believe that the proposed personalized system is feasible, comprehensive, and useful. Certain participants were not convinced that the proposed system would be easy to implement. For instance, a safety manager highlighted the possible high cost associated with implementing the phased approach.

## 5. Conclusions

Given the pervasiveness of technology and the disruptive nature of emerging technologies such as WSDs, there is a critical need for researchers to develop guidance that can encourage the smooth integration of useful technologies into mainstream practice. To tackle the issue surrounding resistance to using WSD for monitoring construction workers’ safety and health, the present study first identified and assessed the different types of WSDs available in the construction industry. Smartphone-based WSDs, smart hard hats, and smart safety vests are the most popular WSDs and the most preferred by FWs. Subsequently, the researchers identified 11 WSD functions—four physiological and seven environmental functions—that are currently available in the industry, followed by an assessment of the impact of each WSD function on construction workers’ safety and health, using insight from 330 construction workers with prior experience using WSDs on construction projects. Results from the study suggest that each function plays an important role in improving workers’ safety and health; however, the “energized cable/equipment detection,” “toxic gas detection,” and “smoke/fire detection” emerged as the most important WSD functions. Approximately two out of three construction workers are willing to share data captured by WSD functions. Finally, the present study proposed and assessed a three-phased personalized WSD framework. Using a phased approach, organizations could first introduce the three fundamental WSD functions—energized cable/equipment, toxic gas, and smoke/fire detection—followed by physiological data collection functions. This could be followed by other environmental functions (such as noise, falling objects, heavy equipment, open trench detection). Initially, these functions do not have to be available to safety personnel. Only workers should have access to this information.

However, the present study has a few limitations that provide opportunities for future research. The present study did not discretize finding, along with demographic factors such as company size and type, work experience, type of trade, safety knowledge, previous accident experience—which are all important variables that could influence the outcome of the study. In certain cases, such as types of trade, available data were insufficient to discretize at the trade level. Moreover, discretizing the present study would significantly impact the length of the present study. Nevertheless, the present study is novel, provides unique findings, and relies on a robust research process. Future research could further discretize the analysis to capture the impact of these demographic variables on workers’ perception of the importance of WSDs and its functions.

Assessing the perspective of workers with no experience using monitoring devices is of equal importance. Given that recent studies suggest that workers’ experience using WSDs could impact the decision to use or perception of effectiveness, future studies should include participants with no prior experience using WSDs. The present study focused on individuals with experience using WSDs to ensure that the data captured are based on actual use, not solely perception. It is believed that capturing such information provides significant benefits and could be useful evidence to convince individuals who are resistant to using WSDs.

While the present study provided a personalized WSD framework, the study did not validate the utility of this framework on live projects. Future studies should develop personalized WSDs using the proposed personalized system framework and test the personalized WSDs on construction projects.

## Figures and Tables

**Table 1:** Wearable sensing devices’ (WSDs) functions and sensor technologies.

WSD Functions		Abbrv.	Sensing Technologies	References
**Physiological Monitoring**				[3,9,11,12,14,18,21,23,24,25,26,31]
F1	Monitor workers body temperature	BodyTemp	Thermistor, infrared, radar	
F2	Monitor workers heart/respiratory rate, and blood pressure	Heart rate monitor (HRM)	ECG/EKG, infrared, radar	
F3	Monitor workers body posture, speed, orientation	BodyPosture	Gyroscope, accelerometer, magnetometer	
F4	Monitor workers stress level	Stress	ECG/EKG, infrared, radar	
**Environmental Sensing**				
F5	Detect toxic gases or chemicals	ToxicGas	Infrared	
F6	Monitors worker proximity to energized cables or high voltage	EnergizedCable	GPS, RFID, UWB, Infrared, Radar, Bluetooth	
F7	Monitors noise level	Noise	Noise sensor	
F8	Detects smoke and compromised air	Smoke/Fire	Infrared	
F9	Detects proximity to equipment or vehicle	HeavyEquip	GPS, RFID, UWB, Infrared, Radar, Bluetooth	
F10	Detect potential falling object		Gyroscope, accelerometer, magnetometer	
F11	Detect open trench excavation	OpenTrench	GPS, RFID, UWB, Infrared, Radar, Bluetooth	

**Table 2:** Demographic Information.

**Education**	**Count**	**%**
High school or less	51	16
Some college up to associate	103	31
College BS	135	41
Advanced degree	41	12
Total	330	100
**Job title**	**Count**	**%**
Construction Manager	106	32
Project Manager	81	25
Carpenter (Drywall installer, roofer, framer/joiner, flooring installer, pile driver, cabin maker/installer, etc.)	42	13
Other FW/Tradesperson	18	5
Equipment operator	16	5
Electrician	16	5
Foreman	14	4
Plumber	14	4
Mechanical Worker (HVAC, insulation, elevator, gas line installer, etc.)	12	4
Mason	6	2
Ironworker (Steel fixer, welder, steel erector, etc.)	5	2
Total	330	100

**Table 3:** WSDs used on construction projects.

Category	Types of WSD	Count (*n* = 330)	%
WSD integrated into personal protective equipment (PPE)	Smart Safety Hard hats (e.g., Cat Detect, Smart CAP)	237	72
	Smart Safety Vests (e.g., Redpoint)	214	65
	Smart Safety Glasses (e.g., XOEye)	205	62
	Smart Safety Boots (e.g., SolePower)	131	40
WSD integrated into typical personal accessories/technologies	Smartphone apps	276	84
	Smartwatch	157	48
	Smartband	109	33
	Smart ring	55	17
	Smart bracelet	62	19
	Smart necklace	39	12
Other WSDs	Wearable lights (e.g., Halo Lights)	211	64
	Attachable devices (e.g., Spot-R Clips, Compass, HMT-1z1)	191	58
	Wearable cameras (e.g., Veho Muvi VCC-005)	132	40

**Table 4:** Summary of the survey results on the importance of WSD functions.

WSD Functions	Total (*n* = 311)			FWs (*n* = 131)			MPs (*n* = 180)			Sig
	Mean	SD	MNV	Mean	SD	MNV	Mean	SD	MNV	
Smoke/Fire detect	5.81	1.43	1.00	5.69	1.59	1.00	5.89	1.29	0.79	0.00
Prox. to energized cable	5.79	1.36	0.88	5.60	1.49	0.70	5.92	1.25	1.00	0.00
Toxic gases or chemicals detect	5.74	1.49	0.59	5.59	1.62	0.67	5.85	1.38	0.50	0.00
Prox. to potential falling object	5.74	1.41	0.41	5.56	1.58	0.57	5.88	1.25	0.71	0.00
Prox. to equipment or vehicle	5.71	1.44	0.17	5.48	1.59	0.30	5.89	1.30	0.78	0.00
Monitor workers body temperature	5.67	1.62	0.12	5.52	1.52	0.43	5.78	1.53	0.00	0.00
Monitor workers HRR/blood pressure	5.66	1.49	0.12	5.46	1.62	0.23	5.80	1.37	0.14	0.00
Monitor noise level	5.66	1.42	0.12	5.43	1.39	0.13	5.83	1.34	0.36	0.00
Prox. to an open trench excavation	5.66	1.49	0.12	5.45	1.65	0.20	5.81	1.34	0.21	0.00
Monitor workers body posture	5.64	1.43	0.00	5.44	1.55	0.17	5.79	1.31	0.07	0.00
Monitor workers stress level	5.64	1.49	0.00	5.39	1.65	0.00	5.82	1.33	0.29	0.00

Sig = One-Sample Statistics; SD = Standard Deviation; MNV = Mean Normalized Value.

**Table 5:** Workers’ Data Sharing Summary.

Metric	FWs (*n* = 131)		MPs (*n* = 183)	
	Only Worker (%)	Worker & Safety Personnel (%)	Only Worker (%)	Worker & Safety Personnel (%)
Monitor workers body temperature	27.48	72.52	38.25	61.75
Monitor workers heart/respiratory rate, and blood pressure	30.53	69.47	29.51	70.49
Monitor workers body posture, speed, orientation	30.53	69.47	29.51	70.49
Monitor workers stress level	26.72	73.28	28.42	71.58
Monitor Toxic gases or chemicals	31.30	68.70	25.68	74.32
Monitors worker proximity to energized cables or high voltage	25.95	74.05	33.33	66.67
Monitors noise level	31.30	68.70	33.88	66.12
Detects smoke and compromised air	30.53	69.47	38.80	61.20
Detects proximity to equipment or vehicle	34.35	65.65	31.15	68.85
Alerts a worker of presence of potential falling object	41.22	58.78	33.33	66.67
Alerts workers close to an open trench excavation	37.40	62.60	31.69	68.31

**Table 6:** Research outcome assessment.

Key Research Finding	Level of Agreement
The most common WSDs used on construction projects are smartphones, smart hard hats, and smart vests.	8.0
The WSDs mostly preferred by construction workers are smartphones, smart hardhats, and smart vests.	6.5
All the identified common WSD functions can play an important role in improving worker safety and health.	7.5
Detection of “Smoke/Fire,” “Proximity to energized cable,” “Toxic gases or chemicals,” and “Potential falling object” can be categorized as some of the most critical WSD functions.	8.0
Workers’ openness to sharing data from the four functions listed above would be helpful to management in improving the safety and health of workers.	9.5
Functions based on environmental sensing and physiological monitoring are equally impactful (on workers’ safety and health).	7.5
There is a higher tendency for management to acknowledge the impact of WSDs functions than fieldworkers.	8.0
Adequacy of research design and process *	8.5
Statistical analysis is adequate *	8.5
Categorizing the functions into environmental and physiological functions is acceptable *	9.0

* Only researchers responded to these questions.

**Table 7:** Personalized WSD system evaluation.

Personalized WSD System Evaluation Criteria	Rating
Feasibility	7.5
Usefulness of functions in the phased system	7.0
Ease of implementation	6.0
Comprehensiveness	9.0
Potential resistance from workers to a personalized system	6.0
Potential resistance from management to the personalized system	5.0

## Data Availability

The data presented in this study are available upon reasonable request from the corresponding author. The data are not publicly available due to privacy restrictions.
